# Leveraging twitter data to understand nurses’ emotion dynamics during the COVID-19 pandemic

**DOI:** 10.1007/s13755-023-00228-9

**Published:** 2023-06-23

**Authors:** Jianlong Zhou, Suzanne Sheppard-Law, Chun Xiao, Judith Smith, Aimee Lamb, Carmen Axisa, Fang Chen

**Affiliations:** 1 Data Science Institute, University of Technology Sydney, Ultimo, Australia; 2 Faculty of Health, School of Nursing & Midwifery, University of Technology Sydney, Ultimo, Australia; 3 Research Office, University of Technology Sydney, Ultimo, Australia

**Keywords:** Mental health, Nurse, Nurse student, COVID-19, Twitter

## Abstract

The nursing workforce is the largest discipline in healthcare and has been at the forefront of the COVID-19 pandemic response since the outbreak of COVID-19. However, the impact of COVID-19 on the nursing workforce is largely unknown as is the emotional burden experienced by nurses throughout the different waves of the pandemic. Conventional approaches often use survey question-based instruments to learn nurses’ emotions, and may not reflect actual everyday emotions but the beliefs specific to survey questions. Social media has been increasingly used to express people’s thoughts and feelings. This paper uses Twitter data to describe the emotional dynamics of registered nurse and student nurse groups residing in New South Wales in Australia during the COVID-19 pandemic. A novel analysis framework that considered emotions, talking topics, the unfolding development of COVID-19, as well as government public health actions and significant events was utilised to detect the emotion dynamics of nurses and student nurses. The results found that the emotional dynamics of registered and student nurses were significantly correlated with the development of COVID-19 at different waves. Both groups also showed various emotional changes parallel to the scale of pandemic waves and corresponding public health responses. The results have potential applications such as to adjust the psychological and/or physical support extended to the nursing workforce. However, this study has several limitations that will be considered in the future study such as not validated in a healthcare professional group, small sample size, and possible bias in tweets.

## Introduction

It has been around 2 years since the outbreak of the novel Coronavirus Infectious Disease 2019 (COVID-19), which was declared by The World Health Organisation (WHO) as a pandemic in March 2020 [1]. COVID-19 has led to an unprecedented impact on people’s daily lives in the past two years infecting 421 million confirmed cases and attributed to more than 5.85 million related deaths globally up until 20th February 2022.^1^ Australia reported over two million confirmed cases with more than three thousand deaths.^2^ The first confirmed case of COVID-19 in Australia was reported on the 25th of January 2020. Cases were linked to overseas travel until community transmission was reported on 7th March 2020 [2]. The virus is highly infectious posing a challenge to contain the spread globally. Many countries have experienced several waves of COVID-19, and witnessed the emergence of different variant strains of COVID-19 (WHO).

Beyond the direct impact of COVID-19 related morbidity, the pandemic has contributed to an increased incidence of distress and mental health-related symptoms. Studies reported increased rates of depression, worry, fear, stress, sadness, disgust, anxiety, bereavement and perceived stigmatisation [3–6] exacerbated by multiple waves of COVID-19 and subsequent public health responses. These mental health issues can also occur in individuals who are not at high risk of getting infected [4]. The mental distress and ill-health at a population level has been attributed to relationship difficulties such as family conflicts, decreased enjoyment or unhappiness of life, problems with drugs, tobacco, and alcohol, suicide or homicide, heart disease, and weakened immune system as well as other medical conditions [7]. Similarly, the detrimental impact that epidemics and/or pandemics have on nurses has been described. Previous international studies post severe acute respiratory syndrome (SARS) [8, 9], Ebola and more recently waves of the COVID-19 pandemic reported nurses who provided direct patient care feared the risk of acquiring and /or transmitting infection, experienced psychological stress, moral distress, and long-term mental health symptoms such as post-traumatic stress disorders, anxiety and depression and emotional exhaustion [10–12].

The Australian nursing workforce being the largest discipline in healthcare have been at the forefront of the COVID-19 pandemic response since the first Australian case was identified. Australian nurses implemented changes to practice and healthcare to address the projected number of intensive care unit (ICU), critical care and hospital beds required. Rapid restructuring of ward spaces, the creation of additional ICU beds, isolation beds (hot, warm, and cold zones), and triage areas was undertaken. Concurrently, the deployment of nursing staff to work in other clinical areas, or to work in COVID-19 specific wards, primary health settings and contract tracing hubs occurred. Anecdotally, the changes Australian nurses experienced were challenging and stressful. The impact of COVID-19 on the Australian nursing workforce, current and future, is largely unknown as is the emotional burden experienced by nurses throughout the different waves of the pandemic and during the subsequent federal and New South Wales (NSW) state based public health responses. Understanding the Australian nursing workforce emotional reactions and responses is central to maintaining a healthy nursing workforce and essential to sustain the fight against COVID-19.

Social media has been increasingly used by people to express their thoughts and feelings during the pandemic period,^3^.^4^ Taking Twitter as an example, it is social by nature, across Twitter feeds and within cultural or other groups such as nursing. Twitter feeds, can be leveraged to understand people’s sentiment and emotion in real-time [13]. Recently, Twitter has been increasingly used as an easy accessible approach to detect mental and emotional disorders [14–16]. Twitter has also been used to analyse mental health issues due to COVID-19 [3, 5, 17]. For example, Li et al. [18] classified tweets into the emotions of anger, anticipation, disgust, fear, joy, sadness, surprise and trust, where two emotions of sadness and fear are more related to severe negative sentiments due to COVID-19. These previous work focus more on public community sentiment and emotion analysis [6]. However, few studies have sought to analyse the emotional dynamics and sentiment of tweets posted by healthcare professional such as nurses during the COVID-19 pandemic. Analysing group level data using deep learning model and generalised framework of six emotions offers a unique approach to explore the emotional dynamics of registered nurses and Bachelor of Nursing degree (BN) students (also called student nurses in this study) during the COVID-19 pandemic. The answers to the questions that we wish to find are:

- How regsitered nurses’ and BN students’ mental health was affected by COVID-19 in the time dimension?
- Can we detect the effects of different waves or variants of COVID-19 on registered nurses’ and BN students’ mental health?
- Can we detect the effects of policies/measures implemented by the government or significant events on registered nurses’ and BN students’ mental health during COVID-19?

This study measured the emotions and the dynamics of nurses and BN student emotions residing in New South Wales (NSW) during the COVID-19 pandemic. The findings have the potential to determine the burden and real-time impact of COVID-19 on nurses parallel to the unfolding epidemiology, the concurrent change to healthcare, federal and state-based public health policy and public health strategy to contain the spread of COVID-19. Findings are likely to have implications for health services and universities engaged to teaching BN students. Understanding which scenarios are associated with negative emotions may be critical to avoid a generation of nurses experiencing poor mental health. Twitter data in the state of New South Wales (NSW) in Australia were collected and analysed to extract emotions which may be affected by COVID-19 and related events during the COVID-19 pandemic. The contributions of this paper primarily include:

- A novel framework with Twitter data is proposed to extract emotion of registered nurses and BN students during the COVID-19 pandemic;
- Instead of the general public community, the emotion of specific groups of registered nurses and BN students are examined to understand how COVID-19 affects their emotions;
- The links between the emotion dynamics of registered nurses/BN students and the development of COVID-19 are investigated;
- The links between the emotion dynamics of registered nurses/BN students and public health measures implemented by the federal and NSW state government during the COVID-19 pandemic are examined;
- The commonly discussed topics during the COVID-19 pandemic are examined and linked to emotion dynamics.

To the best of our knowledge, this is the first study to utilise a computerised deep learning program and modelling to observe the emotional dynamics of registered nurse and BN student groups across the COVID-19 pandemic from January 2020 to September 2021 with the use of Twitter data.

## Related Work

In this section, we review the related work for mental health especially mental health of nurses during COVID-19. We also highlight how our work differs from these existing approaches.

### Social media and mental health

Social media has long been used as the data source for mental health analysis such as sentiment, depression and emotion analysis due to the largely available user-generated text data [19, 20]. The posted text data and the social behaviour of the social network users are assumed to contain clues for identifying mental health such as emotion status. Different approaches are proposed to detect emotions in tweets ranging from classic machine learning techniques to advanced deep learning approaches [20, 21]. Most of the methods take the concept of supervised machine learning for social media emotion analysis [20]. For example, Colnerič and Demšar [22] exploited hashtags to create three emotion-labeled data sets corresponding to different categories of emotions. They found that recurrent neural networks showed better classification performance compared with bag-of-words and latent semantic indexing models. These approaches have also been used in different applications. Wang and Wei [23] analysed emotions expressed in tweets using a deep learning model developed in [22] to understand emotions in the cancer community. Results showed that joy was the most commonly shared emotion, followed by sadness and fear in the cancer community. Vo and Collier [24] detected and tracked emotions of people in earthquake situations with the use of tweet classifications. Srinivasan et al. [25] proposed the use of emotion classification of Twitter data for predicting 2016 US presidential elections. A lexicon-based classifier was used to determine the emotions and the sentiments within the tweets which were then used to predict the swing direction of the 19 US states towards the candidates of the 2016 US presidential election.

These previous work motivates us to use Twitter data to detect nurses’ and nurse students’ emotions. However, our work will further examine dynamics of emotions over different waves of COVID-19.

### Twitter and COVID-19

People’s mental health during the COVID-19 has been extensively investigated in the past two years, however they are mainly on the general community in the state and country levels [3–5]. The impact of COVID-19 on people’s mental health based on Twitter data analysis has been recently investigated in various research. For instance, Ridhwan and Hargreaves [26] analysed people’s emotion during COVID-19 in Singapore with the use of Twitter data. The research found that there were changes in the prevalence of fear and joy emotions during COVID-19, and people showed more joy when they tweeted about staying at home. Medford et al. [27] used Twitter data to understand public emotion at the early stage of COVID-19 outbreak. It was found that approximately half of all tweets expressed fear and around 30% expressed surprise. The most commonly discussed topics in tweets were the economic and political impact of COVID-19. Park et al. [28] analysed the public discourse on COVID-19 in four Asian countries with outbreaks at varying degrees of severity: South Korea, Iran, Vietnam, and India with the use of Twitter. The research found that each government’s official phases of the epidemic were not well aligned with the degree of public attention represented by the daily tweet counts. Valdez et al. [29] conducted a longitude study with the use of Twitter to understand people’s mental health during the COVID-19 pandemic. Topic modelling approaches were used to detect topics discussed in Twitter in different stages. It was found that the topics of tweets in the early months were mostly COVID-19–specific events. However, as state and municipal governments began issuing stay-at-home orders, the topics shifted toward US-related lifestyle changes rather than global pandemic-related events.

Different from these previous work, our work investigates the dynamics of emotions over the COVID-19 period for specific groups and link the development of COVID-19 with the dynamics to identify whether and how the development of COVID-19 affects the mental health. Furthermore, previous work mainly focuses on the mental health of the general public. In contrast, our work aims to understand emotions of specific groups of nurses and BN students during the COVID-19.

## Data

### Study location

This paper focuses on a case study of analysing registered nurses’ and BN students’ emotional responses during the COVID-19 pandemic in the state of New South Wales (NSW) in Australia. NSW is the Australia’s most populated state with around 8.189 million people according to the census in June 2021 from Australian Bureau of Statistics.^5^ Sydney, the NSW’s capital city, is Australia’s most populated city with a population of over 5.367 million.

### Data collection

We collected Twitter data from Twitter users who live in different Local Government Areas (LGAs) of NSW in Australia. The time span of the data is from 1 January 2020 to 30 September 2021 which covers different COVID-19 waves and different variants in NSW as well as various public health actions conducted by the Australian and NSW government. For example, the first confirmed case of coronavirus was reported in NSW (25 January 2020), the first time that the NSW government announced the relaxing for the lockdown policy (10 May 2020), and the first Delta variant case (7 June 2021).^6^

In this study, Twitter data (text data) were collected through the user timeline crawling API *user*_*timeline*^7^ in Tweepy which is a Python wrapper for the official Twitter API.^8^ The description of each Twitter user of collected tweets was also collected. The description was then analysed to find nurse or nurse student related text and identify nurses or nurse students among Twitter users. The analysis was done by using an algorithm to detect nurses and nurse students in the description automatically. Table 1 shows the summary of the collected tweet dataset. In summary, 400 nurse users and 17 nurse student users were identified respectively from the collected 169,528 Twitter users. Each nurse posted around 750 tweets while each nurse student posted around 600 tweets in average over the study period.

**Table 1:** Summary of the collected Twitter dataset

Description	Size
Total Twitter users	169,528
Total nurse users	400
Total tweets from nurse users	300,573
Total nurse student users	17
Total tweets from nurse student users	10,186

**Fig. 1:**
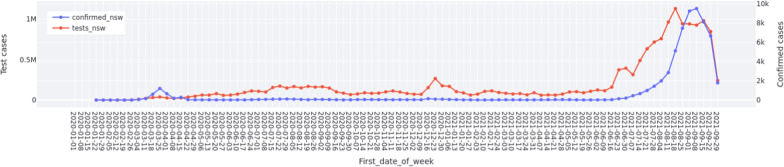
Overview of COVID-19 in NSW from 1 January 2020 to 30 September 2021

Figure 1 shows the weekly overview of the number of test cases (“tests_nsw”) and confirmed cases (“confirmed_nsw”) of COVID-19 in NSW over the study period. These data were collected from DATA.NSW.^9^

## Methodology

**Fig. 2:**
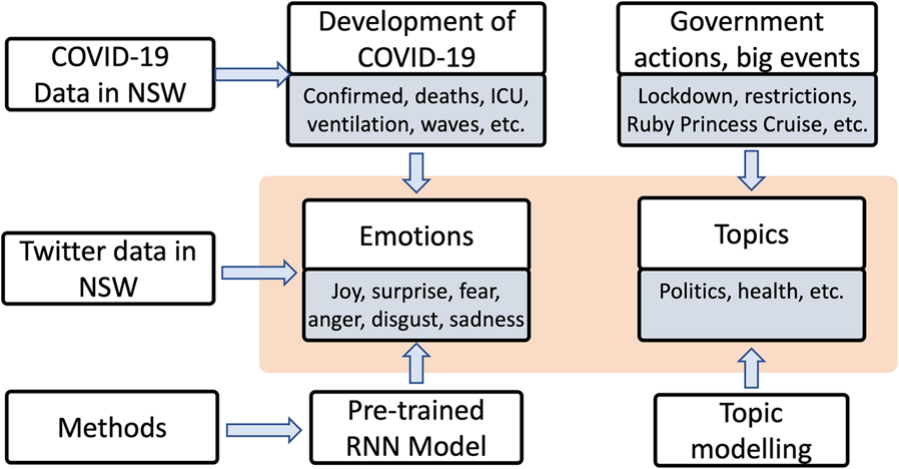
A framework to comprehensively understand emotion dynamics in this study

In this study, the collected tweets are firstly pre-processed such as cleaning and filtering. Nurses’ and BN students’ emotion is then analysed from two perspectives: overall emotion as well as their dynamics. Thereafter, we drill down into lexicon level to understand what words and topics are discussed and concerned by nurses and BN students using topic modelling. The emotion is also linked with government public health policies and other events to find how they affect nurses’ and BN student groups. Figure 2 presents the framework proposed in this study to comprehensively understand emotion dynamics.

### Emotion analysis in tweets

Ekman [30] proposed a set of six universally recognizable basic emotions: anger, disgust, fear, joy, sadness and surprise that differed from each other in prominent manners. This study aims to examine these six basic emotion dimensions from each tweet.

Furthermore, Colnerič and Demšar [22] developed a deep learning algorithm to predict six basic emotions from tweets, which was trained with a data set of 73 billion tweets with a recurrent neural network (RNN) models. The model has been successfully used to detect emotions in tweets by other studies [23]. It also has been used to detect emotions in tweets during the COVID-19 pandemic [26]. This pre-trained RNN model is available via an open-sourced access.^10^ This model is used in our study to classify the tweets into their prevailing emotions.

In this study, a tweet is firstly input into the pre-trained RNN model and get the classified emotion. Since this study aims to analyse weekly emotion of nurses and nurse students as well as their dynamics, emotions of tweets are thereafter aggregated to represent the weekly emotion of nurses and nurse students, which are calculated with the equation:

1 $$\begin{aligned} p_{nurse}&= \frac{N_{nurse}}{N} \end{aligned}$$

2 $$\begin{aligned} p_{nur\_stu}&= \frac{N_{nur\_stu}}{N} \end{aligned}$$

where $N_{nurse}$ and $N_{nur\_stu}$ represent the number of tweets with a given emotion for nurses and nurse students in a week respectively, $p_{nurse}$ and $p_{nur\_stu}$ represent the percentage of tweets with a given emotion for nurses and nurse students in a week respectively, and *N* is the total number of tweets in a week posted by nurses or nurse students.

Moreover, the emotion dynamics is defined as the change rate of the percentage of tweets of positive or negative emotion over a week and is calculated with equations:

3 $$\begin{aligned} r_{nurse}^{i}&= \frac{p_{nurse}^{i} - p_{nurse}^{i-1}}{p_{nurse}^{i-1}} \end{aligned}$$

4 $$\begin{aligned} r_{nur\_stu}^{i}&= \frac{p_{nur\_stu}^{i} - p_{nur\_stu}^{i-1}}{p_{nur\_stu}^{i-1}} \end{aligned}$$

where $p_{nurse}^{i}$ and $p_{nurse}^{i-1}$ are the given emotion of nurses in the current week *i* and the previous week $i-1$ respectively, $p_{nur\_stu}^{i}$ and $p_{nur\_stu}^{i-1}$ are the given emotion of nurse students in the current week *i* and the previous week $i-1$ respectively, while $r_{nurse}^{i}$ and $r_{nur\_stu}^{i}$ are the change rate of a given emotion for nurses and nurse students respectively in the current week *i*.

Table 2 shows examples of tweets from nurses with different emotions classified with the RNN model.

**Table 2:** Examples of tweets with different emotions of nurses

#	Date	Tweets	Emotion
1	27/12/2020	Hey #MTPTproject. Has anyone stayed overnight in hospital without their partner after a c-section? How did that go for you and bub? I had an emergency c-section with our first and really needed my partner, but our now-toddler needs overnight care too	Fear
2	27/12/2020	My workplace previously was not compulsory, still isn’t but now if you don’t have it you can’t work in places like Intensive care even if you’ve been working there for 30 years.. I believe Public hospital is the same-mandatory is a relative term-don’t have to..don’t have to work!	Joy
3	13/08/2021	Open and HONEST communication from ALL levels would help, the ongoing supply to proper PPE helps, the banter and laughs on the floor help. But we can no longer all sit in the tearoom and be inappropriate together which helps in “normal” times	Joy
4	3/09/2021	We’re averaging 3k new cases and around 90 new hospital admissions a day. ICU is at 80% and they’re now beginning to cancel other procedures (before they’ve even caught up from the 30,000 they cancelled last year). Fastest increase in infections is in kids. So, yeah.	Fear
5	3/09/2021	Reporting Exclusive: Covid-19 hospitalisations three times higher than reported	Surprise
6	11/09/2021	Wrong. But even if it were true, so what? Until the hospitalisation number goes down it’s not allowed. That’s the rule	Sadness
7	11/09/2021	I’m not worried that my 11 year old kid will die of COVID, because that’s vanishingly rare. But I would not be happy if he was infected, because he’d then have a 1 in 50 to 100 chance of being hospitalised, which would be shitty especially at the moment	Anger
8	11/09/2021	Doesn’t an increase in covid infections also mean an increase in hospitalisations, especially high cost intensive care? Will governments eventually increase preventative measures to slow increases in health care costs where these are not fully privatised?	Disgust

### Topic modelling

In order to identify the topics that are associated with nurses’ and nurse students’ emotions during the COVID-19 pandemic, the topic modelling method of Latent Dirichlet Allocation (LDA) [31] is used in this study. LDA is a hierarchical Bayesian model and is one of the widely used generative probabilistic topic modeling algorithms. It has also been used by many studies conducting topic modeling on COVID-19 tweets [29].

In this study, an LDA model automatically generates topics from tweets and then groups similar tweets into one or more of these topics based on the distribution of words in tweets. In this study, five topic groups are generated. The top ten high-scoring words in each topic group are selected. Since this study aims to understand what topics are associated with nurses’ and nurse students’ emotions during the COVID-19 pandemic, these high-scoring words from topic groups are then merged to remove potential overlapping words.

## Results

In this section, we firstly present an overview of emotion dynamics of nurse and BN student groups residing in NSW over the study period. Six universally recognized emotions: anger, disgust, fear, joy, sadness and surprise were explored. The emotional dynamics were linked to pandemic related developments to understand how the unfolding pandemic affected emotions and mood. The Australian and NSW state-based government public health measures and significant events (defined as an event that changed the course of the pandemic) are also linked to the pandemic development to find how they affect nurses’ (registered and BN students) emotions. Lastly we examine the topics that both nurse groups discussed to understand what they were mostly concerned with during the pandemic.

### Overview of emotion dynamics in NSW

**Fig. 3:**
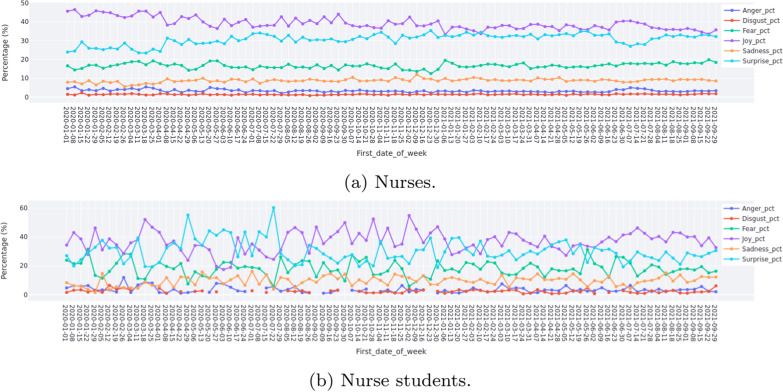
Weekly emotion during the study period

Figure 3a presents the overall weekly emotion dynamics of nurses during the study period. The vertical axis represents the percentage of tweets with one emotion over the whole tweets weekly. From this figure, we can observe that the emotion of joy was dominant with a gradual decline during the study period, while surprise showed an overall increase tread during the study period. Registered nurses’ expression of fear as an emotion fluctuated whilst sadness and anger were less prominent.

Figure 3b shows the overall weekly emotion dynamics of BN students during the study period. Compared with the overall weekly emotion dynamics of nurses as shown in Fig. 3a, BN students expressed a different pattern of emotion. Joy, surprise and fear fluctuated and were more dominant emotions throughout the different pandemic waves. Similar to registered nurses, BN students also showed a degree of sadness, but did not have obvious anger emotion.

### Emotion dynamics and pandemic development in NSW

**Fig. 4:**
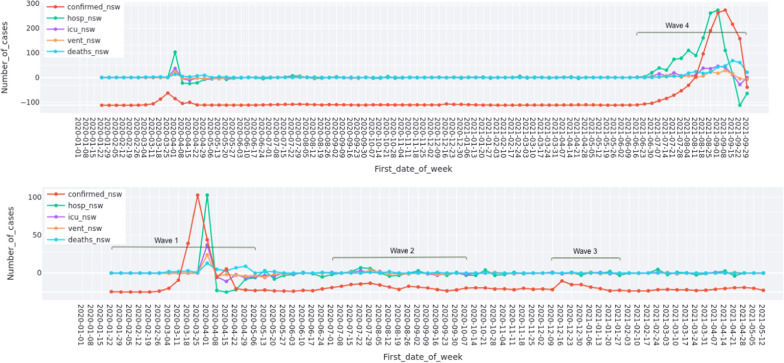
Overview of COVID-19 development in NSW during the study period. (confirmed_nsw: number of confirmed cases; hosp_nsw: number of hospitalisation cases; icu_nsw: number of cases in ICU; vent_nsw: number of cases using ventilation; deaths_nsw: number of deaths.)

This subsection aims to understand whether the development of COVID-19 over time in NSW affected emotions of nurses and BN students during the study period. Figure 4 shows the overview of COVID-19 development in NSW during the study period (the lower figure is the enlarged version of the period before 12 May 2021 in the upper figure). Kapon et al. [32] have identified three waves of COVID-19 in NSW from January 2020 to January 2021. The fourth wave of COVID-19 because of the variant of Delta from June 2021 is added in this study. Therefore, this study analyses changes of emotions of nurses and nurse students in NSW in the following four waves:

- Wave 1: 25 January 2020 — 2 May 2020 (this study focuses on the period between 18 March 2020 and 29 April 2020);
- Wave 2: 1 July 2020 — 7 October 2020;
- Wave 3: 9 December 2020 — 27 January 2021;
- Wave 4: 16 June 2021 — 30 September 2021.

In this study, we examine the correlation between the change rate of different emotions and the development of COVID-19 in four waves respectively.

#### Registered nurses

Table 3 shows the correlation between nurses’ fear change rates and development of COVID-19, where “COVID-19 Data” represents different case numbers of COVID-19, “cor_coef” represents the correlation coefficient between fear change rates and one type of case numbers of COVID-19, “95% CI” represents the 95% Confidence Interval (CI) of correlation coefficients, and “p-value” represents the statistical p value between the change rate of fear and one type of case numbers of COVID-19. From p-values in this table, we did not find any significant correlations between nurses’ fear change rate and the development of COVID-19. However, the confirmed cases (confirmed_nsw) in Wave 1 showed a relatively high positive correlation with the change rate of fear (cor_coef = 0.5628). The number of cases using ventilation (vent_nsw) in Wave 4 also showed a relatively high positive correlation with the change rate of fear (cor_coef = 0.6602). These imply that the change rate of nurses’ fear during the Wave 1 and Wave 4 was highly positively affected by the number of confirmed cases and the number of cases using ventilation respectively.

We also examined other emotions of nurses and found that the change rate of nurses’ disgust was significantly positively correlated with the number of cases in ICU (cor_coef: 0.8445; 95% CI: [0.2511,0.9765]; p<0.0168), and the number of cases using ventilation (cor_coef: 0.7997; 95% CI: [0.1171,0.9691]; p<0.0309) respectively during the Wave 1. It was also found that the change rate of nurses’ disgust was significantly positively correlated with the number of cases in ICU (cor_coef: 0.7236; 95% CI: [0.0387,0.9459]; p<0.0424) during the Wave 3. The change rate of nurses’ surprise was significantly negatively correlated with the number of deaths (cor_coef: $-$0.5563; 95% CI: [$-$0.8316,$-$0.0615]; p<0.0313) during the Wave 2. We also found that the change rate of nurses’ joy was significantly negatively correlated with the number of cases using ventilation (cor_coef: $-$0.8565; 95% CI: [$-$0.9736,$-$0.3830]; p<0.0066) during the Wave 3.

**Table 3:** Correlation between fear change rates of nurses and development of COVID-19

	COVID-19 data	cor_coef	95% CI	p-value
Wave 1	confirmed_nsw	0.5628	[$-$0.3302,0.9242]	0.1883
	deaths_nsw	$-$0.6591	[$-$0.9437,0.1866]	0.1074
	tests_nsw	$-$0.2140	[$-$0.8329,0.6426]	0.6449
	hosp_nsw	$-$0.2264	[$-$0.8368,0.6349]	0.6254
	icu_nsw	$-$0.2232	[$-$0.8358,0.6369]	0.6305
	vent_nsw	$-$0.1284	[$-$0.8037,0.6915]	0.7838
Wave 2	confirmed_nsw	0.0229	[$-$0.4952,0.5290]	0.9354
	deaths_nsw	$-$0.1365	[$-$0.6064,0.4040]	0.6275
	tests_nsw	$-$0.1292	[$-$0.6017,0.4102]	0.6462
	hosp_nsw	0.3395	[$-$0.2092,0.7256]	0.2158
	icu_nsw	0.0941	[$-$0.4393,0.5785]	0.7386
	vent_nsw	0.0645	[$-$0.4630,0.5583]	0.8193
Wave 3	confirmed_nsw	0.3193	[$-$0.4973,0.8359]	0.4408
	deaths_nsw	$-$0.4591	[$-$0.8793,0.3630]	0.2525
	tests_nsw	0.1267	[$-$0.6346,0.7632]	0.7649
	hosp_nsw	$-$0.2351	[$-$0.80620.5628]	0.5752
	icu_nsw	$-$0.1296	[$-$0.7645,0.6329]	0.7597
	vent_nsw	0.6602	[$-$0.0831,0.9315]	0.0748
Wave 4	confirmed_nsw	0.1068	[$-$0.4107,0.5722]	0.6939
	deaths_nsw	0.1166	[$-$0.4024,0.5788]	0.6672
	tests_nsw	0.1647	[$-$0.3605,0.6105]	0.5422
	hosp_nsw	$-$0.0993	[$-$0.5671,0.4169]	0.7145
	icu_nsw	$-$0.0537	[$-$0.5351,0.4541]	0.8436
	vent_nsw	$-$0.0367	[$-$0.5229,0.4675]	0.8928

#### BN students

Table 4 shows the correlation between the fear change rate of BN students and the development of COVID-19. It was found that the change rate of nurse students’ fear was significantly positively correlated with the number of confirmed cases during the Wave 1 (cor_coef: 0.7900; 95% CI: [0.0913,0.9675]; p<.0345), and with the number of cases using ventilation during the Wave 2 (cor_coef: 0.6508; 95% CI: [0.2079,0.8723]; p<.0086) respectively. The change rate of nurse students’ fear was significantly negatively correlated with the number of hospitalisation cases during the Wave 3 (cor_coef: $-$0.7345; 95% CI: [$-$0.9843,$-$0.0618]; p<.0380).

Other types of nurse students’ emotion were also examined and it was found that the change rate of nurse students’ sadness was significantly positively correlated with the number of cases using ventilation during the Wave 2 (cor_coef: 0.5735; 95% CI: [0.0867,0.8392]; p<0.0254). The change rate of nurse students’ surprise was significantly positively correlated with the number of hospitalisation cases during the Wave 3 (cor_coef: 0.7565; 95% CI: [0.1110,0.9531]; p<0.0298).

**Table 4:** Correlation between fear change rates of BN students and development of COVID-19

	COVID-19 data	cor_coef	95% CI	p-value
Wave 1	confirmed_nsw	0.7900	[0.0913,0.9675]	0.0345
	deaths_nsw	$-$0.3270	[$-$0.8666,0.5653]	0.4741
	tests_nsw	$-$0.1271	[$-$0.8033,0.6922]	0.7859
	hosp_nsw	0.2393	[$-$0.6267,0.8408]	0.6053
	icu_nsw	0.3105	[$-$0.5776,0.8620]	0.4979
	vent_nsw	0.2939	[$-$0.5897,0.8572]	0.5223
Wave 2	confirmed_nsw	0.2661	[$-$0.2850,0.6850]	0.3378
	deaths_nsw	0.3155	[$-$0.2347,0.7126]	0.2520
	tests_nsw	$-$0.0753	[$-$0.5657,0.4545]	0.7897
	hosp_nsw	0.1158	[$-$0.4215,0.5929]	0.6811
	icu_nsw	$-$0.1736	[$-$0.6298,0.3717]	0.5361
	vent_nsw	0.6508	[0.2079,0.8723]	0.0086
Wave 3	confirmed_nsw	0.2407	[$-$0.5588,0.8083]	0.5658
	deaths_nsw	$-$0.3641	[$-$0.8505,0.4582]	0.3753
	tests_nsw	$-$0.1712	[$-$0.7816,0.6067]	0.6853
	hosp_nsw	$-$0.7345	[$-$0.9483,$-$0.0618]	0.0380
	icu_nsw	0.1080	[$-$0.6458,0.7552]	0.7991
	vent_nsw	$-$0.1889	[$-$0.7886,0.5950]	0.6541
Wave 4	confirmed_nsw	$-$0.0857	[$-$0.5577,0.4282]	0.7524
	deaths_nsw	$-$0.1524	[$-$0.6026,0.3713]	0.5730
	tests_nsw	$-$0.0355	[$-$0.5220,0.4685]	0.8962
	hosp_nsw	0.0382	[$-$0.4663,0.5240]	0.8883
	icu_nsw	0.0603	[$-$0.4488,0.5399]	0.8245
	vent_nsw	$-$0.0074	[$-$0.5013,0.4901]	0.9782

### Emotion dynamics and government measures and big events

In this subsection, we aim to investigate whether public health interventions undertaken by the federal or NSW state government or significant events (defined as events that changed the course of the pandemic in NSW) affected emotion of registered nurses or BN students. Table 5 shows examples of interventions undertaken by government and events occurred during the study period. Therefore, we analysed the effect of public health interventions undertaken by the federal or NSW state government and significant events (defined as events that changed the course of the pandemic in NSW) on these emotions.

**Table 5:** Examples of interventions undertaken and events occurred during the study period

Date	Description
18 March 2020	The Ruby Princess Cruise docked in Sydney Harbour, which was considered to create a coronavirus hotbed in Australia
20 March 2020	Australian border closure
31 March 2020	NSW lockdown – indoor and outdoor gathering limited to two people in NSW
10 May 2020	The NSW premier announced the relaxing for the lockdown policy
19 December 2020	Restrictions on people in Syndey’s Northern Beaches local government area to contain the spread of COVID-19
20 December 2020	Density restrictions tightened to require 4 square metres per person in indoor areas in greater Sydney
30 December 2020	Limit of 300 people at a hospitality venue and no more than 5 visitors per day for greater Sydney
17 June 2021	The first Delta variant case who was a Sydney driver was reported in NSW

**Fig. 5:**
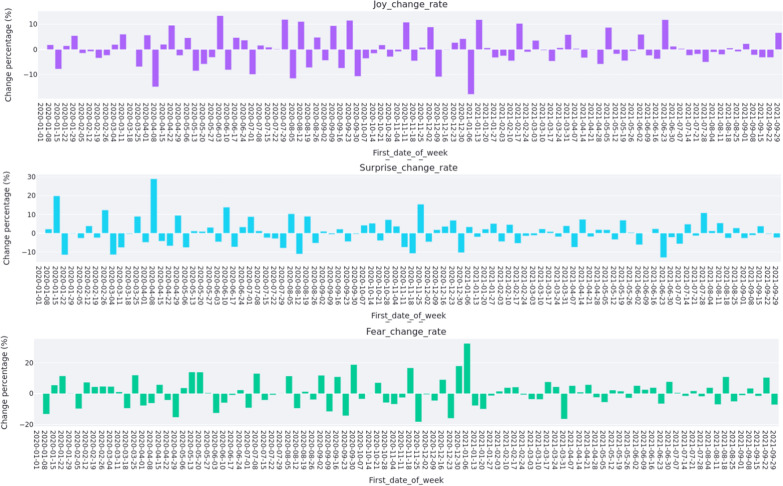
Weekly emotion change rate of nurses

The weekly change rate of joy, surprise and fear for registered nurses are reported in Fig. 5. The Ruby Princess Cruise ship docked in Sydney Harbour on 18 March 2020 with COVID-19 positive cases. Passengers disembarked on 19 March without isolation. Subsequently, 662 confirmed cases and 22 deaths of COVID-19^11^ were linked to the Ruby Princess Cruise Ship. Figure 5 illustrates that registered nurses’ emotions declined in joy and increased in surprise after the week of 18 March 2020 which also covers the date of Australian border closure. Nurses showed a sharp increase in fear after the week of 18 March 2020. On 31 March 2020, NSW officially started the lockdown policy. It was found that nurses’ joy decreased in the week of 31 March 2020 and then increased after this week. Nurses’ surprise and fear increased in the week of 31 March 2020 and then decreased after this week. Nurses’ tweets expressed increased surprise and fear as well as decreased joy between the week of 6 May 2020 and week after, which reflect a relaxing for the lockdown policy announced on 10 May 2020. As shown in Fig. 5, nurses’ joy, surprise and fear did not show much changes in the week of 16 December 2020 which covers the announcement of restrictions for great Sydney. In the week of 30 December 2020 and the week after when new limitations were applied, nurses showed a big decrease of joy, and an increase of surprise and fear. Moreover, minimal change in joy, surprise and fear was found in the week of 16 June 2021, which covers the report of the first Delta variant case observed in Sydney.

**Fig. 6:**
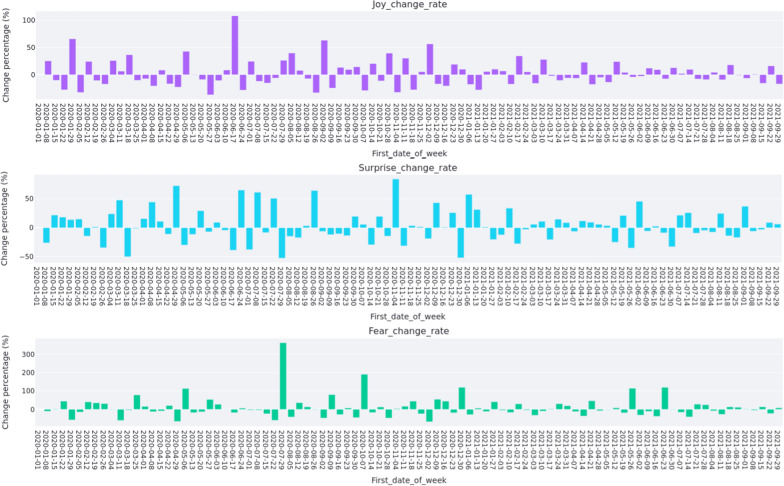
Weekly emotion change rate of nurse students

Figure 6 shows the weekly change rate of joy, surprise and fear of nurse students. From this figure, we can see that in the week of 18 March 2020, there was a small increase in joy and decrease in fear for nurse students, which implies that the Ruby Princess Cruise event and Australian border closure did not affect joy and surprise of nurse students too much. However, nurse students showed a big increase in fear after the week of 18 March 2020. When the NSW lockdown was relaxed on 10 May 2020, nurse students showed a big increase in joy and decrease in surprise, but still had a big increase in fear. During the week of 16 December 2020 when some restrictions were introduced in greater Sydney, BN students did not show much changes in joy, surprise and fear. However, when further restrictions were introduced in the week of 30 December 2020 in great Sydney, BN students showed a big decrease in surprise and big increase in fear, revealing the strict restrictions may be affected emotion of nurse students negatively. When we look at the week of 16 June 2021 when the first Delta variant was reported in Sydney, BN students’ joy, surprise and fear did not change significantly.

### Emotion and tweet topics

This subsection analyses the topics that registered nurses and BN students talked in Twitter during the study period to understand what topics affect their emotions. Table 6 shows the word clouds of tweet topics related to fear in different waves of COVID-19 in NSW for nurses and nurse students. Overall, we can see that COVID was one of major topics in different waves for both registered nurses and BN students. We also drill down into tweets and summarised topics for further insights. It was found that both registered nurses and BN students expressed different talking topics during the COVID-19 waves. For example, in the wave 1, registered nurses’ talk focused on personal protection equipment, lockdown, self-isolation, lies, politics and government and others; nurse students focused topics of health, hospital, and food; but both groups talked about their pets, government, racism, rules, protect/support/avoid, emergency, doctor, scientist, respect, mask, fear, die, death, and lockdown. BN students revealed an overlap of some topics with registered nurses such as aged-care, family, racism, and politics, they also showed different topics such as death, Victorian/Melbourne, isolation, and student. In the wave 3, nurses’ major topics shifted to vaccines, school, emergency, mask, lockdown, and other similar topics as in the wave 2 of politics, racism, and death. Nurse students still talked about Victorian/Melbourne’s wave besides job, staff, education, mask/safe/isolation. In the wave 4, registered nurses tweeted more about support, vaccine, police, politics, ICU, death, safe, hospital, health, risk, NSW, lockdown, PM, community, Afghanistan, kid, and women. While BN students had similar topics with nurses such as lockdown, police, risk, and vaccine, they also revealed topics such as ICU, protest, sick, mask.

The analysis showed that the main topics that both nurse groups discussed on Twitter were on politics, racism, and healthcare. The events occurred during different waves also affected their fear topics in the Twitter. However, BN students had different talking topics from nurses besides common topics. For example, BN students talked more on Melbourne’s several waves of COVID-19 in 2020 and 2021 while nurses did not.

**Table 6:**
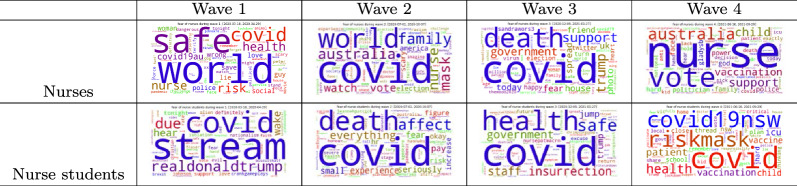
Tweet topics related to fear in different waves of COVID-19 in NSW

Besides fear topics, we also analysed topics affected other emotions such as joy. Similar to the topics related to fear, politics, health and vaccination were talked about, which affected the joy of both nurses and BN students. Furthermore, both nurses and BN students were happy to talk about support related to COVID-19. However, there were differences in topics related to joy between the two groups. For example, nurses talked more about family, community, and Christmas related topics. While BN students talked more about student and school related topics.

Overall, we can see that similar to the general public, both nurse groups talked more about COVID-19, politics, and health. Other topics that were occurred during the COVID-19 pandemic were also commonly discussed, such as racism, family and protest. Different groups had different talking topics, for example, BN students talked more on Melbourne’s waves, while nurses talked more on family and Christmas. Different waves also affected both groups’ topics, such as the personal protective equipment and mask in the early waves and vaccine in the later waves. All these topics affected emotion of nurses and nurse students differently.

## Discussion

To the best of our knowledge, this is the first study that found significant correlations between the emotional dynamics of nurses (registered and BN students) and the unfolding COVID-19 pandemic as well as government public health measures and significant events that occurred in NSW. The tweeted group-level data analyses illustrate the weekly-wise emotional dynamics of nurses (registered and student) and could provide an understanding of the real-time emotional affect of COVID-19. The two nurse groups offer different viewpoints and this may be due to the variation in professional accountability, context and patterns of work and/or study. However, the differences are important to explore to understand the varied emotional responses during the COVID-19 pandemic. The unique approach offers an alternate approach to survey question-based instruments which are often used to understand people’s beliefs [33], and may not reflect actual everyday emotions but the beliefs specific to survey questions. This is a significant difference between a survey-based study and a data-driven approach. The following subsections discuss the emotional dynamics of both groups.

### Joy

Although the emotion of joy is unexpected and in contrast to published international and national qualitative and quantitative studies [12], joy was present across both nurse groups and throughout the study period. The fine-grained weekly analysis may account for the prominence of this emotion detecting the small positive changes to home life, healthcare environment, state-based management and control of COVID-19 cases. Nurses, as a professional group are well informed about public health strategies, infection control practices and disease management and therefore their reactive emotions may reflect their knowledge and experience. After the first week of the NSW lockdown (31st March 2020) during wave 1 (18 March 2020 – 29 April 2020) nurses experienced an increase in the emotion of Joy. This could be attributed to nurses’ relief and/or support of public health responses to control the outbreak.

Joy as an emotion may indicate an appreciation of simple pleasures, such as spending time with family and practicing an attitude of gratefulness during the pandemic and could have contributed to the increase in Joy during this time frame. The acquisition of protective factors such as self-compassion and resilience may be adaptive behaviours over time [34]. Self-compassion entails showing kindness, love and empathy to oneself rather than being overly critical when experiencing adversity. The literature shows that self-compassion was found to be protective against depressive symptoms [35–37] and burnout in nurses [38] and more experienced nurses reported greater self-compassion [39].

### Surprise

Surprise may reflect the anticipated number of COVID-19 related hospitalisations, particularly in contrast to the scale and impact of the COVID-19 pandemic in American and European countries. During wave 1 (18 March 2020 – 29 April 2020) there was minimal change in rate of nurses’ surprise. This could also be credited in part to nurses inherent moral resilience. The literature reports that as nurses mature in the profession so too does their moral resilience; allowing them to maintain perspective, keep a situation in context, and understand that some conditions are out of their control [40, 41]. Nurses during wave 1, may also have felt confidence in the government leadership to manage the situation in response to regular and effective communication [26].

The change rates of nurses’ and BN students’ surprise was significantly increased in wave 2 (1 July 2020 – 7 October, 2020) and wave 4 (16 June 2021 – 30 September 2021). There was a correlation to the relaxing of the lockdown policy announced on the 10th May 2020 and the announcement of mandatory vaccinations for student nurses on the 30th August 2021. The increase in rates of surprise coincided with an increase in fear and a decrease in joy. These changes, although they appear to be related to events, may suggest an increase in moral distress amongst nurses. High levels of emotional exhaustion and burnout in nurses during the COVID19 pandemic and subsequent waves has been widely documented in literature [12]. Moral distress develops when nurses are consistently forced to perform and make decisions in response to the pandemic which run counter to their training and responsibilities [42]. Studies have reported the relationship between moral distress and a variety of mental health problems amongst nurses [40].

### Fear

The accumulated knowledge of how COVID-19 was transmitted and the positive effect of public health measures may have contributed to low levels of constant fear. The emotion of fear was present in both nurse groups throughout the study duration. Despite minimal correlations between the development of COVID-19 and the fear change rates during the four waves respectively, significant correlations between the fear change rate and COVID-19 case numbers and patient ventilation rates were reported. There was a sharp increase in fear for nurses when The Ruby Princess Cruise docked in Sydney Harbour (week of 18 March 2020) indicative of nurses concern and consistent with fear of a potential outbreak. Similarly, registered nurses’ and BN students’ tweets expressed increased fear as relaxing of the lockdown policy was announced on 10 May 2020. The context of fear reported in this study is largely unknown posing an interpretation challenge however the fluctuations of fear as an emotion are present. Previous studies describe fear in the context of COVID-19 acquisition, transmission to family and/or others and discrimination. Fear has been identified as a factor associated with an increased risk of short and long term psychological distress [43].

Similarly, the BN student’s showed a significant increase in fear after the week of 18 March 2020. Swift and frequent changes became the norm for BN academic programs in New South Wales shifting learning from the classroom to online lectures and limited to no clinical placement. The announcement of mandatory vaccinations for BN Students on the 30 August 2021 coincided with an increase in fear amongst BN students. Elevated levels of stress, anxiety, and depression are common symptoms in BN students.The onset of the COVID-19 pandemic has further exacerbated existing issues and new fears.

### Limitations

The study offers a unique and an alternate approach to explore the emotional dynamic and sentiment of nurses, however there are several limitations that should be considered. The data was analysed using a computerised deep learning program and validated model of six universally acknowledged emotions, however, the model has not been validated in a healthcare professional group and therefore may not reflect the nuances of nurses’ work and/or the emotional resilience of the nursing workforce. Although the volume of tweets analysed in this study were substantial, the authors’ acknowledge the small sample size with less than 1 percent of the NSW registered nurse workforce represented. The sample size of BN student group is similarly small and therefore interpretation of the findings are limited (17 nurse students were identified as Twitter users). Findings may not be generalised across nurse populations providing front-line care to COVID-19 infected patients given the sample size and the unknown professional role of the nurse. In addition, the content of tweets may be subject to bias, limited to personal opinion and general public topics of nurses who are engaged in social media out of work hours and censored due to strict social media policies and nurses’ adherence to their professional code of conduct. Therefore insight into specific nursing work or how the delivery of care affects the emotional dynamics of nurses may be limited. Considering the different levels of responsibility and the considerable difference in hours worked (employment versus clinical placement for 4-8 weeks) between registered nurse and BN student group, limited comparison should be made.

## Conclusion and future work

This paper described the emotional dynamics of registered nurse and BN student groups residing in New South Wales who were engaged in social media during the study period. Twitter data provided a unique real-time insight into the emotions and sentiment of the nursing workforce parallel to various pandemic waves, social change, government public health actions and significant events. The analysis of group tweets using a deep learning computerised model may provide key healthcare stakeholders with an opportunity to adjust the psychological and/or physical support extended to the nursing workforce to preserve the current and future generations of nurses. Future studies will explore more advanced emotion classification models and validation of the model within nursing populations to classify the emotional dynamics and the nuances of nursing work.

## Footnotes

1 https://coronavirus.jhu.edu/

2 https://worldhealthorg.shinyapps.io/covid/

3 https://www.insil.com.au/post/significant-increase-in-social-media-usage-under-covid-19-heres-what-that-means-for-businesses/

4 https://www.who.int/news-room/feature-stories/detail/social-media-covid-19-a-global-study-of-digital-crisis-interaction-among-gen-z-and-millennials

5 https://www.abs.gov.au/

6 https://www.abc.net.au/news/2021-06-17/what-is-the-delta-variant-sydney-covid-outbreak-explained/100223048

7 http://docs.tweepy.org/en/latest/api.html#api-reference

8 https://developer.twitter.com/en/docs/api-reference-index

9 https://data.nsw.gov.au/

10 https://github.com/nikicc/twitter-emotion-recognition

11 https://www.theguardian.com/world/2020/may/02/australias-coronavirus-lockdown-the-first-50-days
